# Effects of Propolis Supplementation on Gut Microbiota and Uremic Toxin Profiles of Patients Undergoing Hemodialysis

**DOI:** 10.3390/toxins16100416

**Published:** 2024-09-25

**Authors:** Larissa Fonseca, Marcia Ribeiro, Júnia Schultz, Natália A. Borges, Ludmila Cardozo, Viviane O. Leal, Marcelo Ribeiro-Alves, Bruna R. Paiva, Paulo E. C. Leite, Carmen L. Sanz, Fernanda Kussi, Lia S. Nakao, Alexandre Rosado, Peter Stenvinkel, Denise Mafra

**Affiliations:** 1 Graduate Program in Medical Sciences, Fluminense Federal University (UFF), Niteroi 24033-900, Brazil; nutrilarissa.sfonseca@gmail.com (L.F.); dmafra30@gmail.com (D.M.); 2 Graduate Program in Biological Sciences-Physiology, Federal University of Rio de Janeiro (UFRJ), Rio de Janeiro 20550-170, Brazil; ribeiromarcia.trabalhos@gmail.com; 3 Bioscience Program, Biological and Environmental Science and Engineering (BESE), King Abdullah University of Science and Technology (KAUST), Thuwal, Makkah 23955, Saudi Arabia; junia.schultz@kaust.edu.sa (J.S.); alexandre.rosado@kaust.edu.sa (A.R.); 4 Institute of Nutrition, Rio de Janeiro State University (UERJ), Rio de Janeiro 20550-170, Brazil; nat_borges_@hotmail.com; 5 Graduate Program in Cardiovascular Sciences, Fluminense Federal University (UFF), Niteroi 24033-900, Brazil; ludmilacardozo@id.uff.br (L.C.); bruna.regis.paiva@gmail.com (B.R.P.); 6 Nutrition Division, Pedro Ernesto University Hospital (UERJ), Rio de Janeiro 20550-170, Brazil; vivianeoleal@yahoo.com.br; 7 HIV/AIDS Clinical Research Center, National Institute of Infectology (INI/Fiocruz), Rio de Janeiro 20550-170, Brazil; mribalves@gmail.com; 8 Graduate Program in Science and Biotechnology, Fluminense Federal University (UFF), Niteroi 24033-900, Brazil; leitepec@gmail.com; 9 Department of Basic Pathology, Federal University of Paraná, Curitiba 81530-000, Brazil; lusanz.alarta@gmail.com (C.L.S.); fernandakussi17@gmail.com (F.K.); lia.nakao@ufpr.br (L.S.N.); 10 Division of Renal Medicine, Department of Clinical Science, Technology and Intervention, Karolinska Institutet, 17165 Stockholm, Sweden

**Keywords:** chronic kidney disease, CKD, hemodialysis, microbiome, uremic toxins, propolis

## Abstract

Background: Propolis possesses many bioactive compounds that could modulate the gut microbiota and reduce the production of uremic toxins in patients with chronic kidney disease (CKD) undergoing hemodialysis (HD). This clinical trial aimed to evaluate the effects of propolis on the gut microbiota profile and uremic toxin plasma levels in HD patients. These are secondary analyses from a previous double-blind, randomized clinical study, with 42 patients divided into two groups: the placebo and propolis group received 400 mg of green propolis extract/day for eight weeks. Indole-3 acetic acid (IAA), indoxyl sulfate (IS), and p-cresyl sulfate (p-CS) plasma levels were evaluated by reversed-phase liquid chromatography, and cytokines were investigated using the multiplex assay (Bio-Plex Magpix^®^). The fecal microbiota composition was analyzed in a subgroup of patients (*n* = 6) using a commercial kit for fecal DNA extraction. The V4 region of the 16S rRNA gene was then amplified by the polymerase chain reaction (PCR) using short-read sequencing on the Illumina NovaSeq PE250 platform in a subgroup. Forty-one patients completed the study, 20 in the placebo group and 21 in the propolis group. There was a positive correlation between IAA and TNF-α (r = 0.53, *p* = 0.01), IL-2 (r = 0.66, *p* = 0.002), and between pCS and IL-7 (r = 0.46, *p* = 0.04) at the baseline. No significant changes were observed in the values of uremic toxins after the intervention. Despite not being significant, microbial evenness and observed richness increased following the propolis intervention. Counts of the *Fusobacteria* species showed a positive correlation with IS, while counts of *Firmicutes*, *Lentisphaerae*, and *Proteobacteria* phyla were negatively correlated with IS. Two months of propolis supplementation did not reduce the plasma levels of uremic toxins (IAA, IS, and p-CS) or change the fecal microbiota.

## 1. Introduction

Chronic kidney disease (CKD) promotes metabolic changes, such as inflammation, oxidative stress, and mitochondrial and endothelial dysfunction, which are causes/consequences of intestinal dysbiosis. These mechanisms are interconnected and promote the progression of CKD and an increase in the development of cardiovascular events [1,2].

The gut microbiota performs several crucial functions, including inhibiting the growth of pathogenic bacteria, promoting immune system homeostasis, producing vitamins, metabolizing undigested carbohydrates into fermentable substrates, and generating short-chain fatty acids (SCFAs) that help maintain the function and integrity of the intestinal barrier [1,2,3,4]. In patients with CKD, gut microbiota homeostasis (called eubiosis) can be compromised (termed dysbiosis) by several factors, including decreased renal function, low fiber intake, the use of some medications (mainly antibiotics), metabolic acidosis, and uremia [2,3,4]. Dysbiosis alters the intestinal environment, increasing the production of uremic toxins, such as indole-3 acetic acid (IAA), indoxyl sulfate (IS), and p-cresyl sulfate (PCS), that compromise the integrity of the small intestine barrier, facilitating its translocation to the circulation [2,5,6,7]. In addition, reduced kidney function contributes to the accumulation of toxins in the circulation, where they are associated with inflammation, oxidative stress, and cardiovascular complications due to their pro-oxidant, pro-coagulant, pro-inflammatory, and pro-apoptotic effects on cells of the cardiovascular system [5,8].

Increasing fiber intake, managing animal protein consumption, and utilizing probiotics, prebiotics, or symbiotics have been proposed to modulate the gut microbiota and reduce toxin production [4,9,10]. In addition, many studies have investigated bioactive compounds, such as those in turmeric and other foods, as modulators of gut microbiota and, consequently, their metabolites [1,4,11,12,13].

Propolis is a resinous material that bees create by gathering various plant exudates [14,15]. Studies have demonstrated many biological properties, such as anti-inflammatory and antioxidant effects [16,17,18]. Propolis has also been suggested as a non-pharmacologic therapy to modulate the gut microbiota through its phenolic compounds [19]. Propolis can contain a variety of chemical components, including acacetin, quercetin, chrysin, rutin, luteolin, apigenin, pinocembrin, myricetin, catechin, kaempferol, naringenin, and galangin [14,15].

These polyphenols may modulate the gut microbiota in different ways, such as modifying gut microbiota composition and functionality (e.g., short-chain fatty acids production) through digested-fermented propolis, transforming them into bioactive metabolites that may affect intestinal ecology and biotransforming propolis via bioconversion using certain strains of *Lactobacillus* [1,2,3]. However, some hypotheses are raised, including antimicrobial effects, inhibiting the growth of some bacteria or prebiotic effects, and stimulating the production of beneficial compounds from polyphenol metabolization [20,21,22]. In addition, propolis could reduce inflammatory processes by modulating the gut microbiota since uremic toxins from the gut microbiota are associated with the inflammatory process [19]. To the best of our knowledge, there are no studies about the effects of propolis supplementation on the gut microbiota of patients with CKD. In the present study, we tested the hypothesis that propolis modulates the gut microbiota and reduces uremic toxin production in HD patients.

## 2. Results

After two months of propolis supplementation, 41 patients completed the study, as detailed earlier [17]. The adherence to the intervention was around 60% for each group. Twenty-one patients were allocated in the propolis group (45 ± 12 years, eight men, BMI 22.8 ± 3.7 kg/m^2^, time on HD 68 ± 60 months, Kt/V 1.66 ± 0.44) and twenty in the placebo group (46 ± 14 years, seven men, BMI 24.8 ± 6.8 kg/m^2^, time on HD 44.5 ± 46.5 months, Kt/V 1.7 ± 0.58).

According to the food intake analysis, the mean total energy intake was 1506 (576) kcal/day, 53.2 (23) g of lipids, 0.93 (0.59) g of protein/kg/day, and 13.8 (6.1) g of fiber/day. There was no significant change after the intervention, as shown in Table 1. Light adverse effects were reported during the study, with a few participants in both groups reporting constipation and none reporting allergic reactions. The typical biochemical characteristics were previously published elsewhere [17], and no changes were observed after interventions. All the data concerning medications were published in our previous study [17], and importantly, their prescription remained the same during the intervention time.

### 2.1. Fecal Microbiota

**Figure 1:**
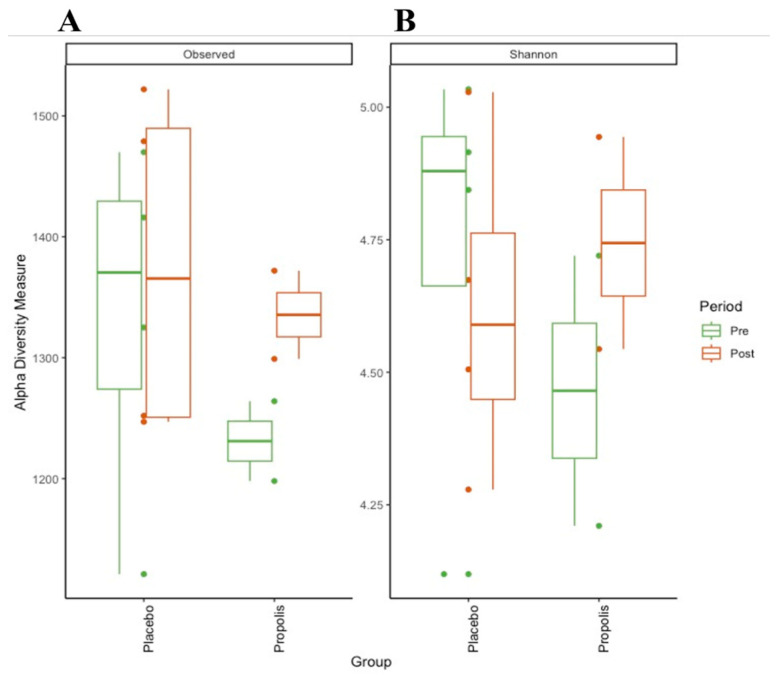
Alpha-diversity of CKD (HD) patients’ gut microbiota on propolis and placebo supplementation. (**A**) Observed zOTUs (richness); (**B**) Shannon index (diversity).

We compared the fecal microbiota in a subgroup of patients (n = 6) grouped in propolis and placebo regarding diversity and their taxonomic composition. The microbial richness (observed number of zOTUs, Figure 1A) and diversity (Shannon index, Figure 1B) of the fecal microbiota in this small group of HD patients showed a non-significant increase in the number of zOTUs after propolis supplementation.

**Figure 2:**
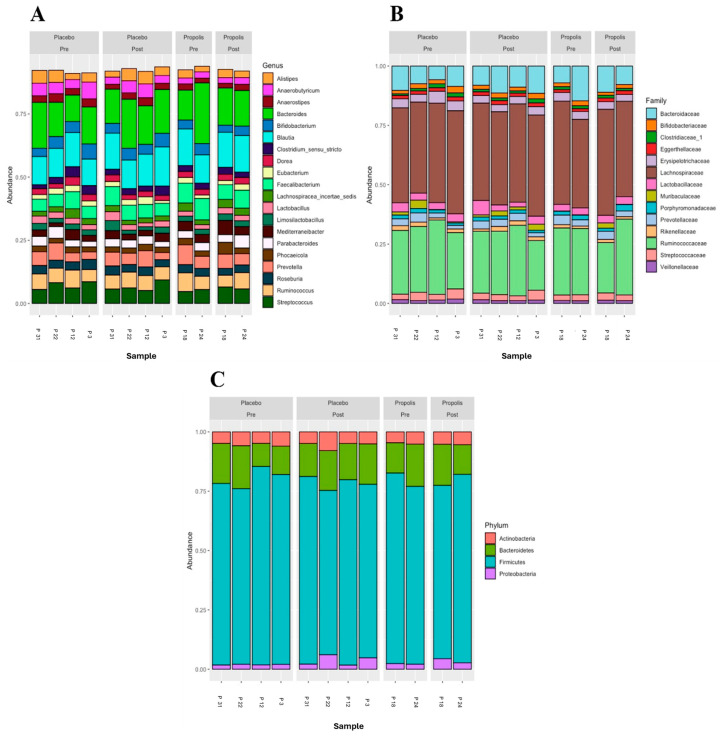
Microbial composition of the placebo and propolis groups in HD patients. Representation at the genus level (**A**), family level (**B**), and phylum level (**C**).

Figure 2 shows the most abundant microbial taxa across six analyzed samples (pre- and post-placebo and propolis of the same patients). The genera *Bacteroides*, *Blautia*, *Faecalibacterium*, *Prevotella*, *Ruminococcus*, and *Streptococcus* were the most abundant in all samples (Figure 2A). Regarding the families, the most abundant taxa in all samples were *Bacteroidaceae*, *Lachnospiraceae*, and *Ruminococcaceae*. Interestingly, the propolis group had an abundance of Muribaculaceae (Figure 2B). Considering the phylum, *Bacteroidetes* and *Firmicutes* were the most abundant in all samples, pre- and post-placebo and propolis, with *Firmicutes* being the most prevalent in both groups and moments (pre and post) (Figure 2C). Comparisons of community composition were performed using beta diversity metrics, and between the groups, this did not show that the microbiota associated with propolis supplementation significantly differed.

### 2.2. Uremic Toxins

**Figure 3:**
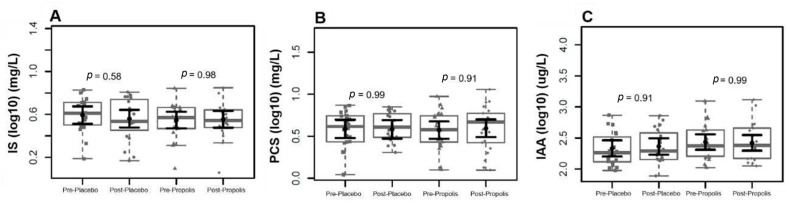
Uremic toxin levels in both groups before and after each intervention. We found no evidence of uremic toxin plasma level differences after two months of intervention in both groups for (**A**) indoxyl sulfate (IS), (**B**) p-cresyl sulfate (PCS), and (**C**) indol acetic acid (IAA). In black, the center circle represents the mean expected marginal effect for each group estimated from linear mixed-effects models. The fixed effects in the models were the intervention group, the time, and its interaction, while the random effect was the patient, and the confounding effects were sex, age, time on hemodialysis, BMI, and Kt/V at the baseline. Black horizontal bars represent the 95% confidence intervals of the expected mean marginal effects by the group. *p*-values were corrected for the number of contrasts/two-by-two comparisons using Tukey’s Honest Significant Difference (HSD) method.

**Figure 4:**
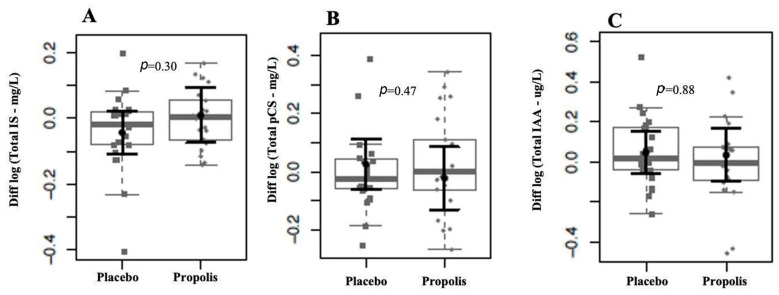
Comparison of uremic toxins between groups after the intervention period. We found no evidence of log-(base 10) fold changes in uremic toxins plasma levels between groups after two months of intervention for IS (**A**), pCS (**B**), and IAA (**C**). In gray, the sample distributions of data are represented in box plots and strip plots. Squares identify the placebo, and circles identify propolis differences in plasma levels between 2 months of treatment and the baseline. In black, the center circle represents the mean expected marginal effect for each group estimated from linear fixed-effects models. The fixed effects in the models were the intervention group, and the confounding effects were sex, age, time on hemodialysis, BMI, and Kt/V ratio at the baseline. Black horizontal bars represent the 95% confidence intervals of the expected mean marginal effects by the group.

**Figure 5:**
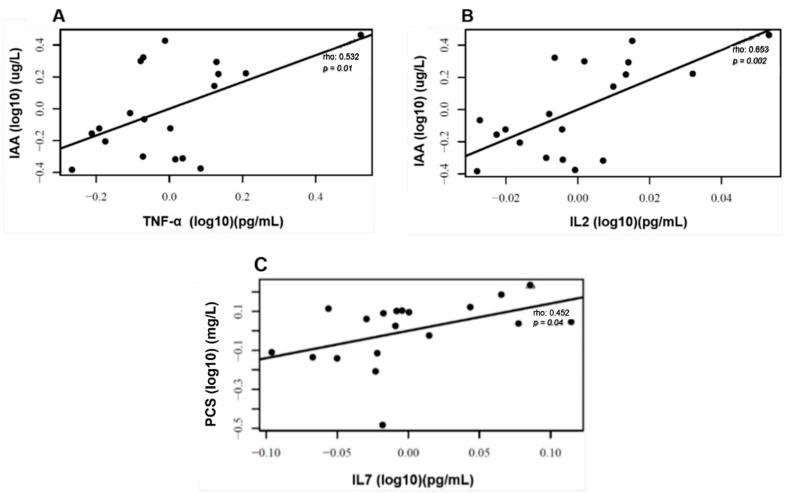
Correlations between uremic toxins and inflammatory markers. Positive correlation at baseline between plasma levels of IAA and TNF-α (**A**), IAA and IL2 (**B**), and PCS and IL7 (**C**). Correlation analyses were conducted using Pearson’s coefficients after adjustments for confounding variables sex, age, time on HD, BMI, and Kt/V ratio.

We observed that the levels of uremic toxins, mainly p-CS and IAA, were higher than the reference values of the European database of toxins (EUTox) (Table 2). None of the baseline data significantly differed between both groups. There were no changes in IS, p-CS, and IAA plasma levels following each intervention (Figure 3). Also, there were no plasma level differences between the two months of treatment and the baseline in the uremic toxins, as shown in Figure 4. Although no significant results were found regarding the reduction in uremic toxins in the plasma, we could observe positive correlations between their levels and the levels of inflammatory cytokines at the baseline, as shown in Figure 5.

### 2.3. Correlations between Fecal Microbiota Composition and Uremic Toxins

**Figure 6:**
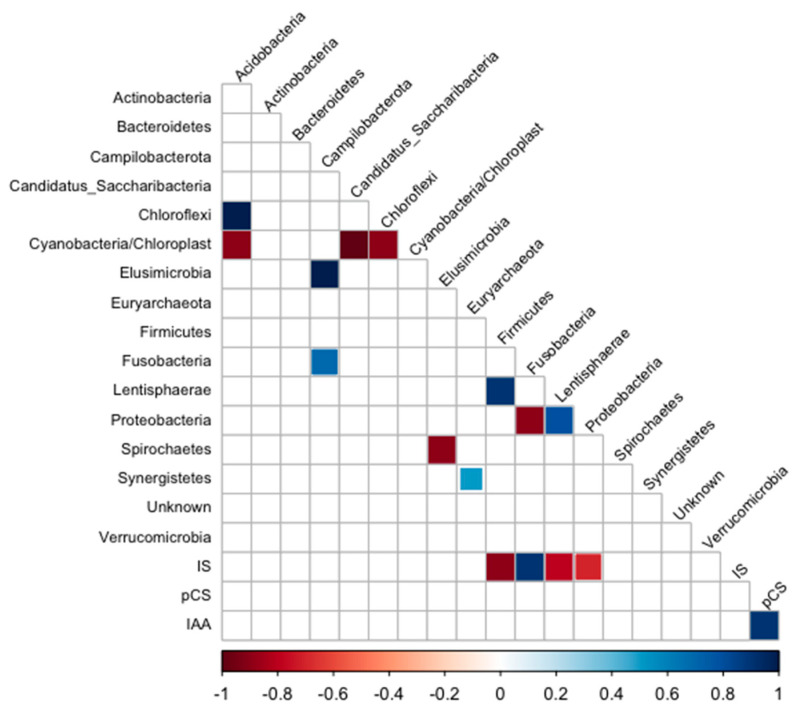
Correlogram of phylum and uremic toxin plasma levels of HD patients. Only significant (*p* < 0.05) positive (blue) and negative (red) correlations are shown in the graph.

The correlogram (Figure 6) shows a positive correlation between IS and *Fusobacteria* and a negative correlation between IS and the bacteria belonging to *Firmicutes*, *Lentisphaerae*, and *Proteobacteria*.

## 3. Discussion

In this secondary analysis of a randomized clinical trial [17], we did not notice a significant difference in the taxa (alpha-diversity) richness after two months of propolis supplementation (400 mg/day). Also, we observed that two months of propolis did not change the plasma levels of uremic toxins that the gut microbiota produced.

Only a few experimental studies have shown positive effects of propolis on gut microbiota modulation. In a survey of colitis model animals, Wang et al. (2017) observed that the supplementation of 0.1%, 0.2%, and 0.3% of Chinese propolis for 21 days modulated the composition of the gut microbiota [23]. The same group of authors in the same animal model observed that supplementation with Brazilian and Chinese propolis (300 mg/Kg) for 17 days modulated the composition of the gut microbiota, reducing *Bacteroides* spp. populations and increasing the diversity and richness of gut microbiota populations [24]. Even though they did not assess plasma levels of uremic toxins, modulating the gut microbiota composition may lead to reduced production since there is a connection between gut microbiota and polyphenols [25]. The gut microbiota transforms polyphenols and phenolic compounds, making them bioavailable. In turn, polyphenols and their metabolites can modulate the gut microbiota and its composition. Furthermore, polyphenols may improve intestinal barrier function by upregulating the expression of genes responsible for producing tight junctions [25,26].

A discussion of how the concentration of propolis may influence the gut microbiota is needed. Previous studies have shown divergences regarding different concentrations and significant changes in the gut microbiota. Cai et al. (2020) evaluated the effects of the ethanol extract of propolis in different concentrations (1% and 2% of ethanol) in mice. They observed significant differences in phyla, bacterial taxa, order, family, and species level, with significant differences in the group with higher concentration [27]. Zheng et al. (2020) observed that a group of mice treated with 150 mg/kg of Chinese propolis did not change their gut microbiota diversity. However, when using 300 mg of propolis/kg, they observed a significant modulation in microbiota diversity, increasing the richness [28].

On the other hand, a study on rabbits receiving different concentrations of propolis (250 and 500 mg/kg) showed a significant reduction in *E. coli* and *Salmonella* spp. in both propolis groups [29]. Beyond altering the gut microbiota composition, propolis may also change the production of its metabolites. Garzarella et al. (2022) observed increased short-chain fatty acids (SCFAs) production, especially acetic and propionic acid after 5 g of dry propolis extract was used in vitro gastrointestinal digestion for approximately 4 h [30].

One study in a CKD animal model observed that Indian propolis extract supplementation reduced uremic toxin plasma levels. In contrast, the animal group not receiving the propolis extract increased the p-CS and IS plasma levels [31]. Moreover, Indian propolis improved interstitial fibrosis and suppressed the expression of α-smooth muscle actin (αSMA) and collagen deposition in the extracellular matrix. This also interrupted the transforming growth factor beta (TGF-β) cascade, decreasing fibrosis processes. These processes represent a vicious cycle between fibrosis and uremic toxins—the more fibrosis, the more retention of these toxins, and vice versa. Since the production of uremic toxins promotes fibrosis [31], it could be an attractive target for patients in the early stages of CKD.

The levels of uremic toxins also need attention. In the present study, the basal plasma levels of p-CS and IAA were higher than the values reported in the European Uremic Toxin (EUTox) database. Although we did not observe a direct effect of propolis on uremic toxins plasma levels, the uremic toxins were correlated with inflammatory markers, which are primary drivers of disease in HD patients. The primary cause of death is cardiovascular diseases, which are closely related to chronic inflammation and dysbiosis [8,32,33].

Indeed, studies have shown the relationship between uremic toxins and the increase in inflammatory markers, which is explained by the aryl hydrocarbon receptor activation (AhR) [5,34,35]. The activation of AhR is associated with inflammation and oxidative stress in the organism by activating the nuclear factor-kB (NF-kB) signaling pathway, which promotes the production of inflammation markers, such as tumor necrosis factor (TNF) and other cytokines [5,35,36,37]. Rossi et al. (2014) observed correlations between IS and TNF, interleukin-6 (IL-6), and gamma-interferon (IFN-γ) in CKD patients [38]. The cross-sectional study of Borges et al. (2016) showed that IL-6 and MCP-1 plasma levels positively correlated with IS, p-CS, and IAA in HD patients [39]. Stockler-Pinto et al. (2018) also showed a positive correlation between uremic toxins and oxidative stress and inflammation markers, such as NF-kB mRNA expression, malondialdehyde (MDA), and C-reactive protein (CRP) plasma levels in HD patients [40].

In this study, we found a positive correlation between IS and the Gram-negative saccharolytic anaerobic bacilli Fusobacteria, which is involved in infection and susceptibility to antimicrobial agents [41,42]. In accordance, this phylum was found in the majority of type 2 diabetes patients with nephropathy. A negative correlation was found between IS and *Lentisphaerae,* a beneficial phylum associated with sleep quality [43]. Still, it was positively associated with type 2 diabetes in the Maskarinec et al. (2021) study [44] and with CRP and LDL cholesterol [45,46]. Gut dysbiosis in CKD increases the abundance of pathobionts, such as *Proteobacteria*, *Enterobacteriaceae*, and *E. albertii*, associated with high uremic toxins levels [47]. Studies show that dietary changes could be beneficial in changing this pathobiont profile in healthier gut microbiota, thus reducing the production of uremic toxins in that environment. Di Iorio et al. (2019) observed that increased levels of *Blautia*, *Faecalibacterium*, *Coprococcus*, and *Roseburia* species are associated with fewer serum toxins in the uremic milieu [47].

Regarding diversity, experimental studies have shown that propolis modulates the gut microbiota composition, leading to increased microbial diversity. A study by Guan et al. (2023) showed that administering propolis to mice significantly altered the gut microbiota, notably increasing the abundance of beneficial bacteria, including *Lactobacillus* and *Bifidobacterium* species [48]. This shift towards a more diverse and balanced microbial community is crucial, as gut microbiota diversity is often associated with better health outcomes and resilience against diseases. Studies highlighted that propolis supplementation in a mouse colitis model increased microbial diversity and ameliorated colitis symptoms, suggesting a protective role against gut inflammation [49,50]. The increase in gut microbiota diversity has significant health implications, such as improved metabolic functions, strengthened immune responses, and a reduced risk of chronic diseases, such as obesity, diabetes, and inflammatory bowel disease (IBD) [49,50].

A limitation of this study is that many factors alter the gut microbiota [51], and the small sample size can compromise the statistical evaluation and the conclusions. Also, the dose and period used in this study may not be the best to modulate gut microbiota and, consequently, cause the production of uremic toxins. Propolis is also known for its antimicrobial action, and this may have been one of the limiting factors in this study since the production of uremic toxins is closely related to the composition of the gut microbiota [30,52,53], and maybe a lower dosage and a lower treatment period could be more attractive in thinking about microbiota modifications. On the other hand, this is one of the few studies relating to propolis in HD patients, mainly to assess plasma levels of uremic toxins and microbiota composition, and a higher dose could significantly change the diversity, reducing the production of uremic toxins. The discussion about the effects of propolis on gut microbiota is in its infancy, and more studies are needed.

In conclusion, the present study showed that a two-month propolis supplementation (400 mg/day) in patients undergoing hemodialysis did not reduce plasma levels of uremic toxins or alter the gut microbiota profile. However, additional data are necessary to properly evaluate these patients’ gut microbiota’s evenness and richness.

## 4. Materials and Methods

### 4.1. Patients

The randomized, double-blind, placebo-controlled clinical trial included patients with CKD on HD from the Centro de Nefrologia Mageense (CENEFRO), Magé, Rio de Janeiro, Brazil, from January to March 2021. The actual study is a secondary analysis of our previous research [17]. The study was approved by the Ethics Committee of the Medicine Faculty of UFF-Niteroi, Rio de Janeiro-Brazil (number 4.449.525) and registered in the clinical trials service of the US National Institutes of Health (NCT04411758).

### 4.2. Inclusion and Non-Inclusion Criteria

Patients with an arteriovenous fistula (AVF) aged between 18 and 75 years old, with CKD on HD for at least six months and with dietary prescription (adequate energy supplying 25–30 Kcal/ideal Kg/day and protein from 1.0 to 1.2 g/ideal kg/day according to the recommendation by NKF-KDOQI 2020) were included in the study. Non-inclusion criteria were patients on HD for <six months, those aged < 18 years, those who tested positive for COVID-19, those using a catheter, those allergic to a bee sting, pregnant patients, smokers, patients who received antibiotics in the last three months, antioxidant supplements, pre, pro or symbiotic supplements, or those with cancer, diabetes, autoimmune and infectious diseases, liver disease and acquired human immunodeficiency syndrome (AIDS).

### 4.3. Study Design

**Figure 7:**
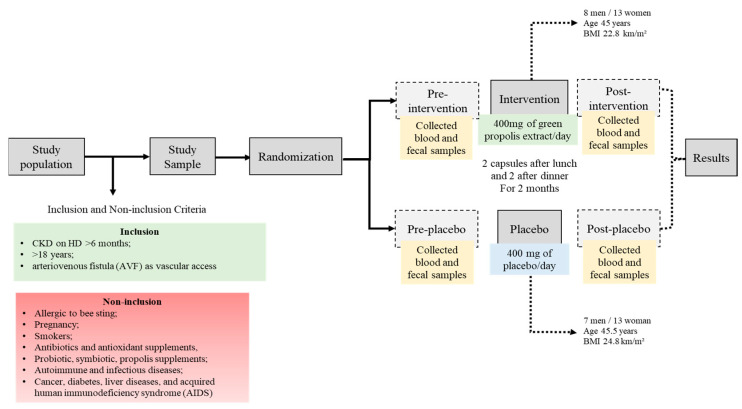
Diagrammatic representation of the study design.

Patients were randomized into pairs by an external party based on matching criteria such as gender, age, body mass index (BMI), and the duration of hemodialysis (HD), following verification of inclusion and exclusion criteria. Eligible patients were then assigned treatment codes in a 1:1 ratio according to a computer-generated list. Patients were then assigned to either the propolis group, which received capsules with propolis extract, or the placebo group, which received capsules containing magnesium stearate, microcrystalline cellulose, and colloidal silicon dioxide. In this double-blind trial, the capsules were labeled as A or B. Participants were instructed to take two 100 mg capsules twice daily, after lunch and dinner, for two months, amounting to a daily total of 400 mg of propolis. This dosage was chosen based on prior research in CKD patients that confirmed its safety [22]. The placebo group received 400 mg/day of microcrystalline cellulose, magnesium stearate, and colloidal silicon dioxide. Blood sample analyses, food intake assessments, and anthropometric measurements were conducted both before and after the supplementation period with propolis extract or a placebo, as illustrated in Figure 7.

Adherence to the supplementation was monitored via telephone contact to ensure that patients were taking the capsules as instructed. At the end of the two-month supplementation period, we counted the remaining capsules in the bottles. Each patient’s adherence percentage was then calculated by dividing the number of remaining capsules by the total number of capsules and multiplying by 100.

### 4.4. Food Intake and BMI Analysis

A 3-day food recall was used to assess the patient’s food intake. Trained nutritionists collected this information on the dialysis day, one day without HD, and one day at the weekend, before and after the two months of supplementation. Dietbox^®^ (version 7.0.21) software was used to calculate food intake. The weight post-HD and height were used to calculate the BMI.

### 4.5. Blood Sample

The HD center-trained professionals collected blood before and after the interventions. The samples were collected in the morning before the dialysis procedure and immediately after the AVF puncture in Vacutainer^®^ tubes containing ethylenediaminetetraacetic acid (EDTA) as the anticoagulant. The time for blood processing after a blood draw ranged from 1 to 2 h. For obtaining the plasma, blood was immediately centrifuged at 1300× *g* for 10 min at 4 °C and separated in aliquots in 1.5 mL polypropylene Eppendorf^®^ tubes, which were identified for each further analysis, and stored at −80 °C. Albumin, glucose, phosphorus, potassium, total cholesterol, and hemoglobin were analyzed in the serum using commercial kits from BioClin^®^ and are presented in the previous study [17].

### 4.6. Fecal Samples

Fecal samples from a subgroup of patients (n = 6) were collected before and after the patients’ interventions after receiving a universal collector and an explanation of how to collect them. Patients were instructed to store the collected fecal samples in a home freezer immediately after collection to preserve the microbial composition. The feces were stored for around 8 to 10 h and did not undergo freeze–thaw cycles. The samples were frozen in the laboratory at −20 °C until analysis.

### 4.7. Uremic Toxin Analysis

Total plasma concentrations of Indole-3 acetic acid (IAA), indoxyl sulfate (IS), and p-cresyl sulfate (p-CS) were determined by reverse-phase high-performance liquid chromatography (HPLC) with fluorescent detection.

Plasma samples were processed, as described by Meert et al. (2012) [54]. Briefly, 100 μL of plasma was diluted with water to 360 µL and heated (95 °C, 30 min). After 10 min in ice, samples were centrifuged at 1300× *g* for 20 min at 4 °C, and the supernatant was ultrafiltered with a 30 kDa cutoff membrane (Amicon Ultra, Millipore, Darmstadt, Germany). Ultrafiltrate (10 μL) was injected. This method has been previously validated [40].

After the process of the plasma, as described previously by Meert et al. (2012), ultrafiltrate was injected into the HPLC system (Shimadzu Prominence, Kyoto Japan) [54]. Chromatographic determinations were performed with a Shimadzu Prominence system equipped with a quaternary pump (Shimadzu LC-20AD), controlled by LC Solution software (version 1.25 SP5), a fluorescence detector (Shimadzu RF-20A), and an autosampler (Shimadzu SIL10-AF). Separation was achieved with a 150 × 4.6 mm, 5 µm, C8 Luna column (Phenomenex), eluted with 50 mM ammonium formate pH 3.0 and methanol, whose proportion increased from 35 to 70% at a flow rate of 0.7 mL/min. During the run, the fluorescence wavelengths varied as follows: λ_exc_ = 280 nm and λ_em_ = 383 nm to IS and IAA (de Loor et al., 2009) and λ_exc_ = 265 nm and λ_em_ = 290 to p-CS [54].

### 4.8. Inflammation Markers

Before analysis, plasma was stored at −80 °C for one month. The plasma levels of the following cytokines were analyzed: monocyte chemotactic protein 1 (MCP-1/CCL-2), macrophage inflammatory protein-1β (MIP-1β/CCL-4), interleukin-2, -6, -7, -8 (CXCL8), -10, and -17, as well as tumor necrosis factor (TNF). This analysis used a multiplex bead-based assay according to the manufacturer’s recommendations, with the Bio-Plex Magpix kit and apparatus (Biorad Laboratories Inc., Hercules, CA, USA). The xMAP magnetic technology used in this assay employed microspheres to detect multiple circulating proteins in the sample, as detailed by Chermut et al. [17].

### 4.9. Fecal Microbiota Sequencing and Analysis

Following the manufacturer’s instructions, DNA was extracted from fecal/soil microbes using the Quick-DNA Fecal/Soil Microbe DNA Miniprep Kit (Zymo Research, Tustin, The USA). The final DNA concentrations were measured using spectrophotometric quantification with NanoDrop 2000 (Thermo Fisher Scientific, Waltham, MA, USA).

A total of six samples (four from the placebo group and two from the propolis group) had their V4 region of the 16S rRNA gene amplified by PCR using primers 515F (5′-GTGYCAGCMGCCGCGGTAA-3′) and 806R (5′-GGACTACNVGGGTWTCTAAT-3′), which were appended with universal Illumina tags. The PCR protocol included an initial denaturation at 94 °C for 3 min, followed by 32 cycles of 45 s at 94 °C, 1 min at 50 °C, and 90 s at 72 °C, with a final extension of 10 min at 72 °C. The resulting amplicons were barcoded, pooled, and sequenced on the Illumina NovaSeq PE250 platform (0.1 M raw reads per sample) according to the manufacturer’s guidelines at Novogene (Commerce, CA, USA).

The 16S rRNA gene amplicon sequencing reads were pre-processed with the USEARCH (v. 11) pipeline, which involved removing primers, filtering, and assembling paired ends. Unique sequences were then used to calculate abundances and cluster Operational Taxonomy Units (OTUs) at 97% identity. Following this, a denoising step was performed to filter out potential PCR chimeras, resulting in a zero-radius OTU (zOTU) table. Feature, taxonomy, and metadata tables were exported as phyloseq objects for further analysis in RStudio version 4.1.2. To ensure comparability between groups, the data were rarefied for subsequent analyses. Community alpha diversity, measured as richness (observed number of zOTUs) and evenness (Shannon index), was computed from 16S rRNA gene zOTU counts using the vegan library (v. 2.6-4). Beta diversity was assessed using Bray–Curtis and weighted UniFrac dissimilarities based on relative zOTU abundances with the phyloseq library (version 1.44.0). The statistical significance of community grouping by the experimental group (placebo or propolis) within the Bray–Curtis and UniFrac spaces was determined using PERMANOVA (via the ‘adonis’ function from vegan). All plots and graphs were created in RStudio using ggplot2 version 3.4.0 or TBtools librarie version No.2.007. The bioinformatics pipeline, including scripts for figure reproduction, is publicly available through the GitHub repository at <https://github.com/juniaschultz/propolis_HD>, accessed on 22 July 2024. Any additional information required to reanalyze the data reported in this paper are available from the lead contact upon request.

## 5. Statistical Analysis

Baseline demographic and clinical continuous numerical variables were compared using non-parametric Mann–Whitney U tests. Uremic toxins and cytokine values were log-transformed (base 10) where necessary and used as outcome measures. Time–intervention interactions were evaluated with multiple linear mixed-effects models, considering patients as a random effect. The fixed component of these models was adjusted for confounding factors such as sex, age, time on HD, BMI, and dialytic efficiency estimated by the Kt/V ratio. Ninety-five percent confidence intervals were calculated, and results were presented graphically to show the estimated mean marginal effects. Mean marginal values were calculated from model parameters, with all factors except the exposition/group effect held at their mean values or equal proportions. Contrasts were derived from these mean marginal effects. *p*-values were adjusted for multiple comparisons using Tukey’s Honest Significant Difference (HSD) method, with statistical significance set at *p*-values ≤ 0.05. Redundancy and Pearson correlation analyses were conducted using R version 4.12.1 with the ‘microViz’ package. Multiple linear mixed-effects model analyses were performed with R version 4.2.1 using the ‘lme4’ and ‘emmeans’ packages and their dependencies.

## Figures and Tables

**Table 1:** Dietary intake of placebo and propolis groups before and after each intervention.

Parameters	Placebo		*p*-Value	Propolis		*p*-Value
	Pre	Post		Pre	Post	
Energy (kcal/kg/d)	24.1 (20.8; 27.4)	21.9 (18.5; 25.2)	0.37	22.0 (17.9; 26.1)	22.5 (18.4; 26.6)	0.98
Protein (g/kg/d)	1.14 (0.94; 1.34)	1.04 (0.83; 1.25)	0.76	0.83 (0.58; 1.08)	0.96 (0.70; 1.21)	0.69
Carbohydrates (g/d)	222 (181; 263)	189 (148; 231)	0.33	226 (175; 277)	212 (160; 263)	0.90
Lipids (g/d)	51.8 (44.5; 59.2)	47.0 (39.6; 54.5)	0.53	45.5 (36.4; 54.5)	55.4 (46.2; 64.6)	0.10
Fiber (g/d)	18.1 (14.3; 21.9)	15.1 (11.3; 19.0)	0.42	13.1 (8.42; 17.8)	14.8 (10.0; 19.6)	0.86

Data are presented as the median (95% confidence interval—CI95%).

**Table 2:** At the baseline, plasma levels of uremic toxins in the placebo and propolis groups.

Uremic Toxins	EUTox	Overall (n = 41)	Placebo (n = 20)	Propolis (n = 21)	*p*-Value
IAA (ug/L)	1005 ± 702	2088 (2396)	1836 (1818)	2374 (2622)	0.09
IS (mg/L)	37.0 ± 26.5	27.7 (16.7)	30.8 (18.9)	27.3 (16.6)	0.24
p-CS (mg/L)	23.0 ± 16.9	28.9 (28.3)	31.3 (27.7)	27.7 (28.3)	0.77

Data are presented as median (interquartile range—IQR) or absolute (relative) proportions. *p*-values were calculated using Mann–Whitney U tests for continuous numerical variables. Abbreviations: IAA: indole-3 acetic acid; IS: indoxyl sulfate; and p-CS: p-cresyl sulfate.

## Data Availability

The original contributions presented in the study are included in the article, further inquiries can be directed to the corresponding author.
